# Fundamental concepts and the latest evidence for esophageal pressure monitoring

**DOI:** 10.1186/s40560-023-00671-6

**Published:** 2023-05-22

**Authors:** Tatsutoshi Shimatani, Miyako Kyogoku, Yukie Ito, Muneyuki Takeuchi, Robinder G. Khemani

**Affiliations:** 1 Department of Emergency and Critical Care Medicine, Graduate School of Biomedical and Health Sciences, Hiroshima University, 1-2-3 Kasumi, Minami-ku, Hiroshima-shi, Hiroshima, Japan; 2 Department of Intensive Care Medicine, Osaka Women’s and Children’s Hospital, 840 Murodo-cho, Osaka Izumi, Japan; 3 Pediatric ICU, Department of Anesthesiology and Critical Care Medicine, Children’s Hospital Los Angeles, 4650 Sunset Blvd., CA Los Angeles, USA; 4 Department of Pediatrics, Keck School of Medicine, University of Southern California, Los Angeles, CA 1975 USA; 5 Department of Critical Care Medicine, National Cerebral and Cardiovascular Center, Suita, Osaka Japan

**Keywords:** Transpulmonary pressure, Esophageal pressure, ARDS, VILI

## Abstract

Transpulmonary pressure is an essential physiologic concept as it reflects the true pressure across the alveoli, and is a more precise marker for lung stress. To calculate transpulmonary pressure, one needs an estimate of both alveolar pressure and pleural pressure. Airway pressure during conditions of no flow is the most widely accepted surrogate for alveolar pressure, while esophageal pressure remains the most widely measured surrogate marker for pleural pressure. This review will cover important concepts and clinical applications for esophageal manometry, with a particular focus on how to use the information from esophageal manometry to adjust or titrate ventilator support. The most widely used method for measuring esophageal pressure uses an esophageal balloon catheter, although these measurements can be affected by the volume of air in the balloon. Therefore, when using balloon catheters, it is important to calibrate the balloon to ensure the most appropriate volume of air, and we discuss several methods which have been proposed for balloon calibration. In addition, esophageal balloon catheters only estimate the pleural pressure over a certain area within the thoracic cavity, which has resulted in a debate regarding how to interpret these measurements. We discuss both direct and elastance-based methods to estimate transpulmonary pressure, and how they may be applied for clinical practice. Finally, we discuss a number of applications for esophageal manometry and review many of the clinical studies published to date which have used esophageal pressure. These include the use of esophageal pressure to assess lung and chest wall compliance individually which can provide individualized information for patients with acute respiratory failure in terms of setting PEEP, or limiting inspiratory pressure. In addition, esophageal pressure has been used to estimate effort of breathing which has application for ventilator weaning, detection of upper airway obstruction after extubation, and detection of patient and mechanical ventilator asynchrony.

## Introduction

Ventilator-induced lung injury (VILI) is an important complication of mechanical ventilation, and in the last several decades we have had an increasing focus on methods to prevent VILI. One of the hallmark physiologic concepts which underpins the risk for VILI is lung stress, which reflects the pressure across the alveoli, or transpulmonary pressure. While there has always been a focus on limiting airway pressure (i.e., plateau pressure or driving pressure), the transpulmonary pressure takes into account the mechanical properties of the chest wall, and therefore provides the best surrogate for the sheer stress across the alveoli [1].

To calculate transpulmonary pressure, both alveolar pressure and pleural pressure must be estimated. The most widely accepted surrogate for alveolar pressure is airway pressure at end-inspiration or end-expiration during an airway occlusion maneuvers. While there are limitations to using airway pressure as a surrogate for alveolar pressure, it is widely accepted in clinical practice. Direct measurement of pleural pressure is invasive and impractical for clinical use. Esophageal pressure has long been used as a less invasive surrogate for pleural pressure. This allows estimation of transpulmonary pressure by calculating the difference between esophageal pressure and airway pressure, frequently measured at end-inspiration or end-expiration:

$${\text{Transpulmonary pressure }}\left( {P_{L} } \right) \, = {\text{ Airway pressure }}\left( {{\text{Paw}}} \right) \, {-}{\text{ Esophageal pressure }}\left( {{\text{Pes}}} \right).$$

As the lung is inflated, transpulmonary pressure becomes positive, either through increase in the airway pressure through the application of positive pressure, or from the generation of negative pleural (esophageal) pressure with spontaneous breathing (or a combination of both during spontaneous and assisted modes of ventilation). While we have often focused on limiting inspiratory positive pressure provided by mechanical ventilation as a method to minimize transpulmonary pressure, we are increasingly recognizing that patients with acute respiratory failure often have strong spontaneous respiratory effort due to hypoxemia, hypercapnia, and hyperinflammatory conditions [2], which generates intense negative swings in pleural pressure with resultant high transpulmonary pressure [2]. In the presence of underlying lung injury, this negative pressure also increases the transvascular pressure, exacerbates pulmonary edema, and causes further injury. The constellation of findings is sometimes referred to as patient self-inflicted lung injury (P-SILI) and suggests the importance of monitoring pleural pressure, and potentially reducing excessive respiratory effort [3].

This review article will focus on important concepts and applications related to the use of esophageal pressure as a surrogate for pleural pressure, with a particular focus on how to use the information from esophageal manometry to adjust or titrate ventilator support to reduce the risks of VILI and P-SILI.

## “How to measure it”

### Measurement of esophageal pressure

**Fig. 1:**
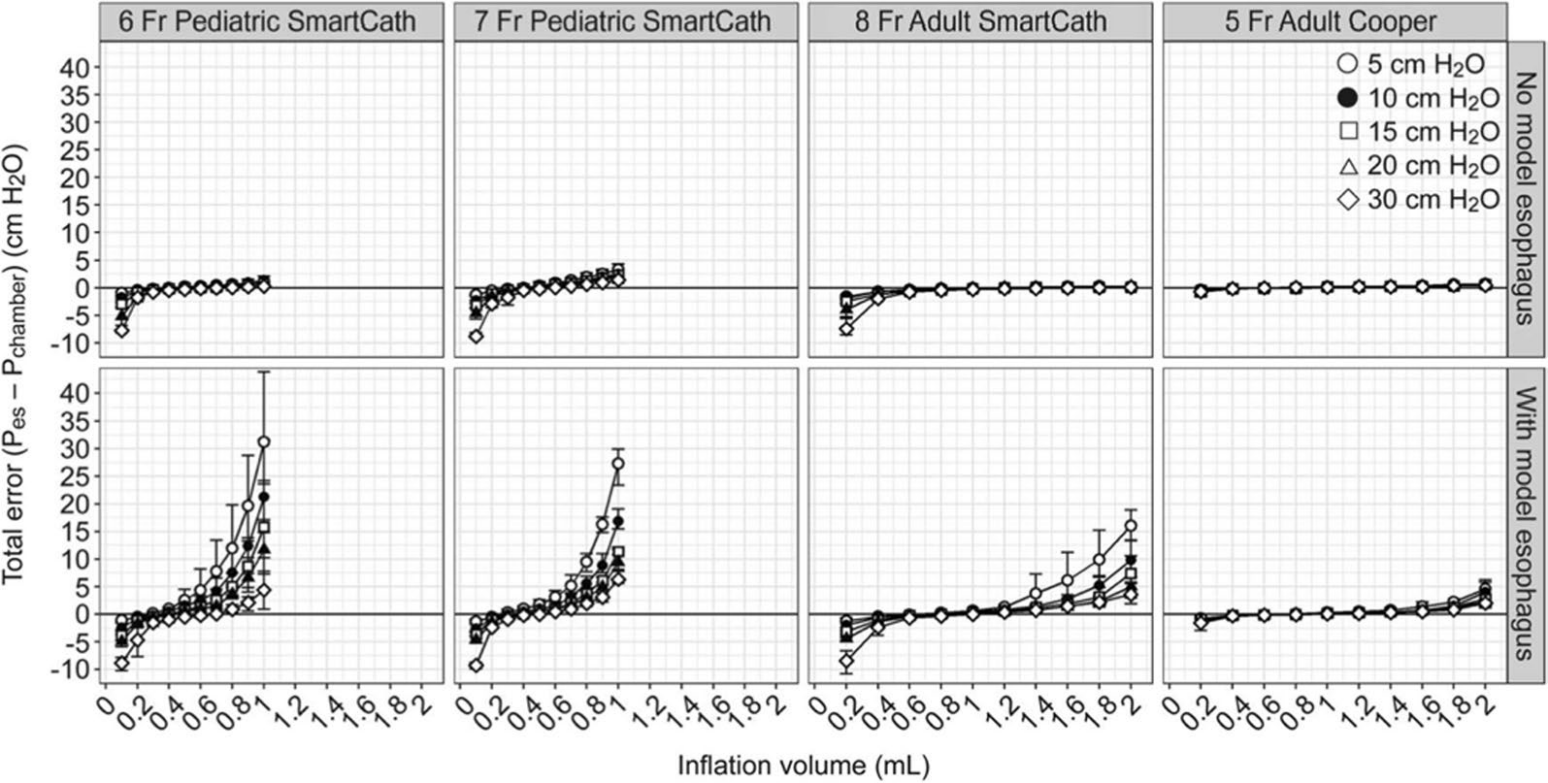
A bench study was performed with pediatric to adult-sized esophageal balloon catheters. Even within the manufacturer's recommended balloon inflation ranges, the impact is greater in children, making it very important to identify the optimal balloon volume. Used with permission of Daedalus Enterprises Inc, from Measurements Obtained From Esophageal Balloon Catheters Are Affected by the Esophageal Balloon Filling Volume in Children With ARDS, Justin C Hotz, 63(2), 2018; permission conveyed through Copyright Clearance Center, Inc

**Fig. 2:**
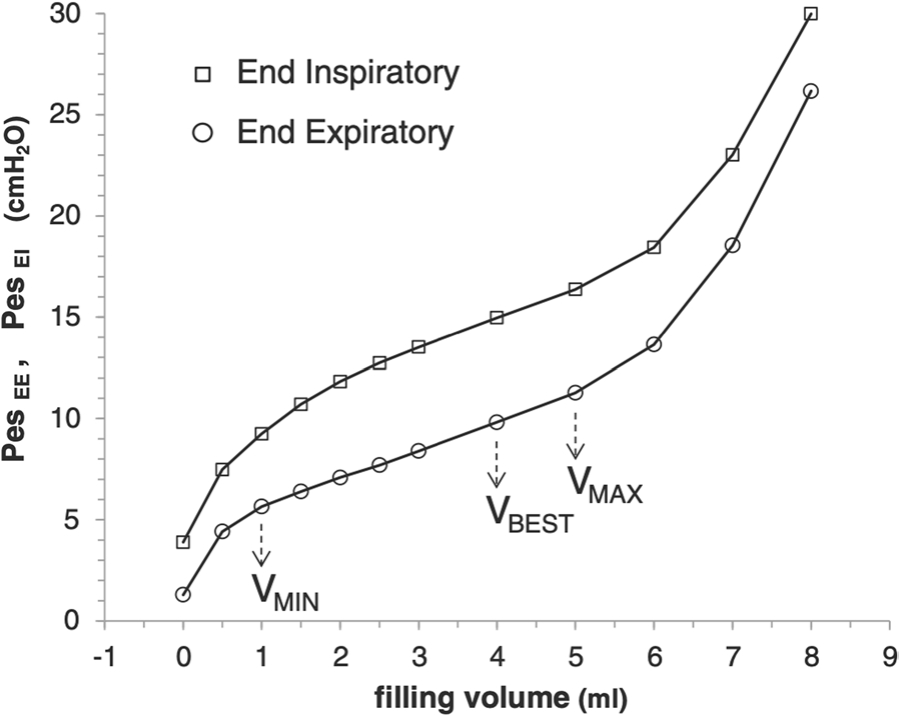
Pressure–volume curve between balloon filling volume and Pes at the end of expiration and inspiration, respectively. Permitted to reprint from [6]. The intermediate linear section was graphically detected and analyzed for its lower and upper limits (*V*_min_ and *V*_max_). Within the appropriate filling range, we identified Vbest, i.e., the filling volume associated with the maximum difference between ΔPes

Methods for measuring esophageal pressure include microtip pressure transducers, and liquid-filled catheters in some cases, but air-filled balloons are the most widely available and used [4]. However, the measurement of esophageal pressure using esophageal balloon catheters is affected by the volume of air in the balloon. Therefore, it is important to calibrate the balloon appropriately and inflate it with the correct amount of air, especially when measuring in small children. If the balloon is under-filled, the pressure transducer within the balloon will under-read. In contrast, if there is too much air in the balloon, then the pressure from the wall of the esophagus can be transmitted through the balloon to the transducer. Hence, the correct balloon inflation volume is strongly affected by the elasticity of the esophagus. Figure 1 shows the results of a bench study performed using pediatric-to-adult-sized esophageal balloon catheters. If there is insufficient air in the balloon, the end-expiratory transpulmonary pressure is underestimated, and if there is too much air in the balloon, the end-expiratory transpulmonary pressure is overestimated. These inaccuracies are seen even when operating within the manufacturer's recommended balloon inflation ranges for both adult and pediatric catheters [5]. Mojoli et al. reported one method to determine the optimal filling volume. [6] Fig. 2 shows the curves expressing the individual pressure–volume (PV) relationship between the balloon filling volume and Pes at the end of expiration and inspiration. It is divided into three phases, depending on the amount of air in the balloon, with the middle zone indicating appropriate inflation volume.

**Fig. 3:**
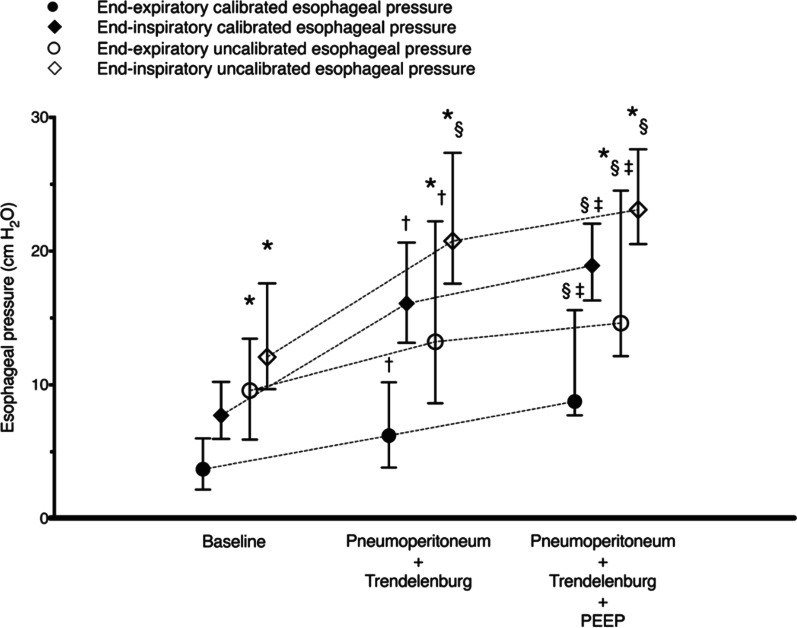
Comparison of calibrated and uncalibrated esophageal pressure values. Applying permission to reprint from [7]. The uncalibrated values were higher than the calibrated values for both the inspiratory and expiratory end esophageal pressure levels

Mojoli et al. also reported a comparison of calibrated and uncalibrated esophageal pressure values (Fig. 3) [7]. The calibration itself can be performed in approximately 10 min with familiarity, although it is important for the patient to have a consistent respiratory pattern from breath to breath or tolerate end-inspiratory occlusions. Esophageal pressure values obtained without calibration are unreliable and may have adverse effects when used to assess or titrate respiratory support.

An alternative method for balloon calibration was proposed by Hotz et al. and focused primarily on deriving a similar esophageal balloon elastance curve focused primarily on the end-expiratory values for esophageal pressure and again solving for a point early in the middle phase of the elastance curve. This method has been demonstrated to be accurate in both adults and children and can be used even when patients have varying degrees of spontaneous effort from breath to breath [8].

**Fig. 4:**
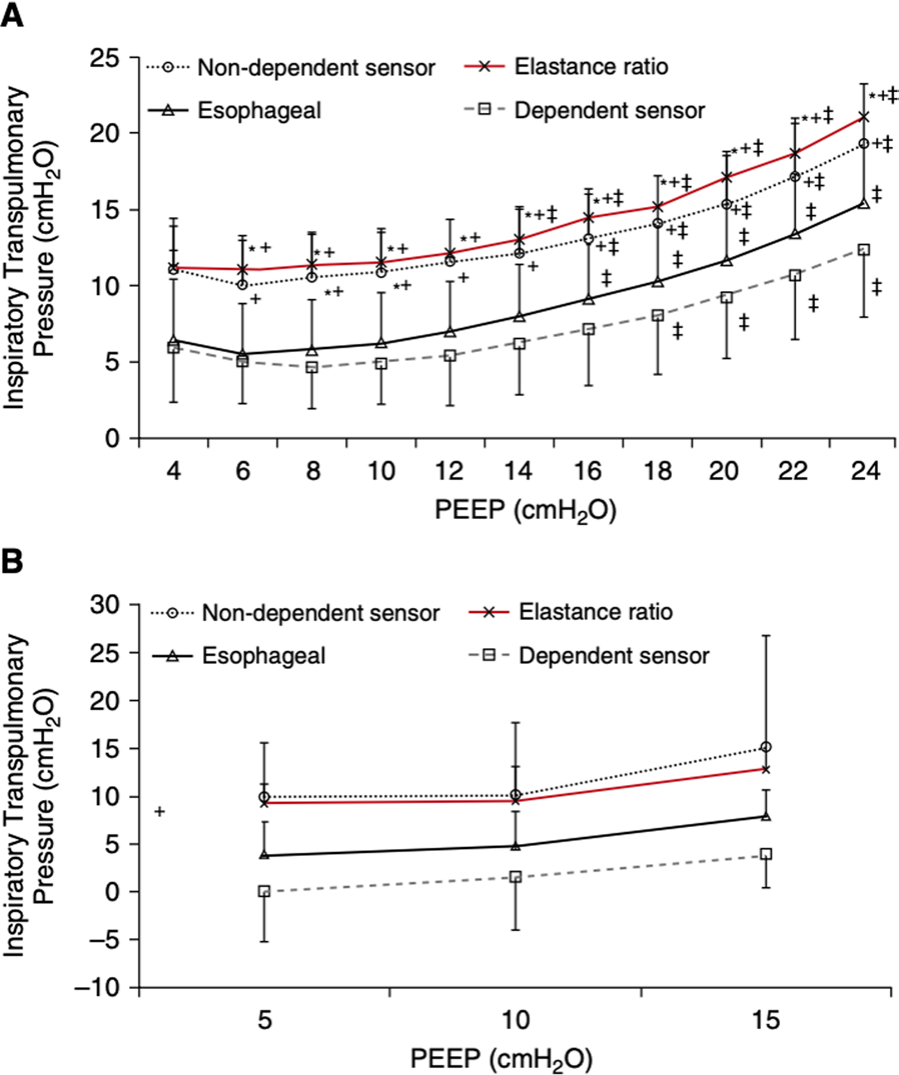
Ppl directly in the dependent and non-dependent regions in lung-injured pigs (**A)** and human cadavers (**B)** and compared the results with those estimated by esophageal pressure. Reprinted with permission of the American Thoracic Society. Copyright © 2022 American Thoracic Society. All rights reserved. Cite: Takeshi Yoshida /2018/Esophageal Manometry and Regional Transpulmonary Pressure in Lung Injury. Am J Respir Crit Care Med, 197(8):1018–1026. The American Journal of Respiratory and Critical Care Medicine is an official journal of the American Thoracic Society. The transpulmonary pressure calculated using the elastance approach reflects the pressure in the non-dependent region, whereas the transpulmonary pressure determined using the direct method reflects the pressure in the area adjacent to the esophageal balloon

When calibrated correctly, balloon catheters give a reliable estimate of pleural pressure, although this reflects the pressure over a certain area within the thoracic cavity. Pleural pressure is regional, and varies stepwise from ventral to dorsal and cranial to caudal. As such, there may be significant differences in regional pleural or transpulmonary pressure, which are either over- or under-estimated based on esophageal pressure [9]. Yoshida et al. measured Ppl directly in dependent and non-dependent regions in lung-injured pigs and human cadavers and compared the results with those estimated using esophageal pressure (Fig. 4) [9]. They found that the transpulmonary pressure determined by the direct method (see below) reflects the pressure in the region close to the esophageal balloon (dependent region), whereas the transpulmonary pressure determined by the elastance method (see below) reflects the pressure in the non-dependent region. Some have proposed that the transpulmonary pressures obtained by the direct method can be used to adjust the PEEP to recruit atelectatic lung units in the dependent region, whereas the transpulmonary pressures obtained by the elastance method can detect the maximum stress at inspiratory pressure.

### Direct method

In the direct method, advocated by Talmor et al., transpulmonary pressure is calculated as the absolute difference between airway and esophageal pressures at end expiration or end inspiration [10]. This direct method is often used at end-expiration to help guide positive end-expiratory pressure (PEEP) titration. At end-expiration (ideally with an expiratory hold to ensure auto-PEEP is accounted for):

$$P_{L} \left( {{\text{end}} - {\text{expiratory}}} \right) \, = {\text{ PEEP }} - {\text{ Pes}} - {\text{PEEP}}{.}$$

When using the direct method to estimate lung stress (end-inspiratory transpulmonary pressure), one must perform an end-inspiratory hold (i.e., measure a plateau pressure):

$$P_{L} \left( {{\text{end}} - {\text{inspiratory}}} \right) \, = {\text{ Pplat }} - {\text{ Pes}} - {\text{plat}}{.}$$

When the direct method is used to estimate lung stress, the *P*_*L*_ at end-inspiration is influenced by *P*_*L*_ at end-expiration. If *P*_*L*_ at end-expiration is very negative (i.e., PEEP is set lower than Pes PEEP), the estimated stress on the lung (i.e., *P*_*L*_ end-inspiratory) will be lower than what is estimated by the elastance method (see below). Proponents of the direct method believe this provides the most appropriate global marker of lung stress throughout the lung.

### Elastance method

The elastance method, proposed by Gattinoni et al. estimates *P*_*L*_ end-inspiratory as the product of plateau pressure and the ratio of the lung to respiratory system elastance [11]:

$${{P}}_{{{L}}} {\text{end}} - {\text{inspiratory}} = {\text{Pplat}}*{\text{E L/Ers}}{.}$$

The ratio of lung to respiratory system elastance is estimated from the change in pressure in the airway and esophageal pressure during tidal ventilation (i.e., from PEEP to the end of inspiration). Because the tidal volume is the same, it cancels out of the equation:

$${\text{E L }}/{\text{Ers}} = \left[ {\left( {{\text{Pplat }} - {\text{ PEEP}}} \right) \, - \, \left( {{\text{Pes}} - {\text{plat }}{-}{\text{ Pes}} - {\text{PEEP}}} \right)} \right] \, / \, \left( {{\text{Pplat }} - {\text{ PEEP}}} \right).$$

Recall driving pressure = Pplat-PEEP. Proponents of the elastance method believe that this is the most appropriate way to estimate *P*_*L*_ end-inspiratory because it is not directly influenced by *P*_*L*_ end-expiration and how PEEP is set, and appropriately accounts for regional differences in transpulmonary pressure (such as the likely pressure in the ventral portion of the lungs which may be at highest risk of lung stress). They believe this therefore reflects the theoretical maximal pressure across susceptible portions of the lungs at end-inspiration.

## "What we can estimate from it"

### Differentiating lung from chest wall compliance

Compliance (*C*) is defined as the change in volume (*V*) associated with a change in pressure (*P*) and is a term used to describe how easily the lungs inflate. Compliance is the mathematical reciprocal of elastance (the change in pressure required to change the lungs by a given volume: *E*). Specifically, static compliance is measured in the absence of flow to eliminate the influence of airway resistance on the pressure required in the respiratory system. Static respiratory system compliance (CRSstat), is usually calculated with parameters obtained from the ventilator and is expressed by the following equation:

$${\text{CRSstat}} = \Delta {\text{V}}/\left( {{\text{Pplat }} - {\text{PEEP}}} \right)$$

$$\left( {{\text{compliance }} = {\text{ change in volume}}/{\text{change in pressure}}} \right).$$

Compliance is inherently different for the lung and chest wall, although respiratory system compliance does not distinguish between them. The pressure applied to the airways by positive pressure ventilation is divided into pressure to expand the lung and pressure to expand the chest wall.

For example, in generalized edema or abdominal compartment syndrome, the same pressure applied to the airways requires more pressure to expand the chest wall, resulting in decreased respiratory compliance, even if lung compliance remains unchanged. As described above, the pressure applied to the lungs (*P*_*L*_) is the most relevant for risk of lung injury. In ARDS, elastance ratios have been reported to vary from case to case [12]. Therefore, the same airway pressure may result in very different transpulmonary pressures from patient to patient. This highlights the importance of estimated lung and chest wall elastance to guide selection of ventilator settings when respiratory system compliance is decreased. Using esophageal pressure, it is possible to assess compliance of the lungs and chest wall.

The following equation calculates lung compliance (Clung):

$${\text{Clung}} = \Delta {\text{V }}/ \, \left[ {\left( {{\text{ Pplat }} - {\text{ PEEP }}} \right) \, - \, \left( {{\text{ Pes}} - {\text{Plat }} - {\text{ Pes}} - {\text{PEEP }}} \right)} \right].$$

The following equation calculates chest wall compliance (Ccw):

$${\text{Ccw}} = \Delta {\text{V }}/ \, \left( {{\text{Pes}} - {\text{Plat }} - {\text{ Pes}} - {\text{PEEP }}} \right).$$

The relationship between respiratory system compliance (CRSstat), lung compliance (Clung), and chest wall compliance (Ccw) is expressed by the following equation:

$${1 }/{\text{ CRSstat }} = { 1 }/{\text{ Clung }} + { 1 }/{\text{ Ccw}}.$$

### PEEP and atelectrauma

PEEP is an essential component of ventilator management, particularly in patients with ARDS.

**Fig. 5:**
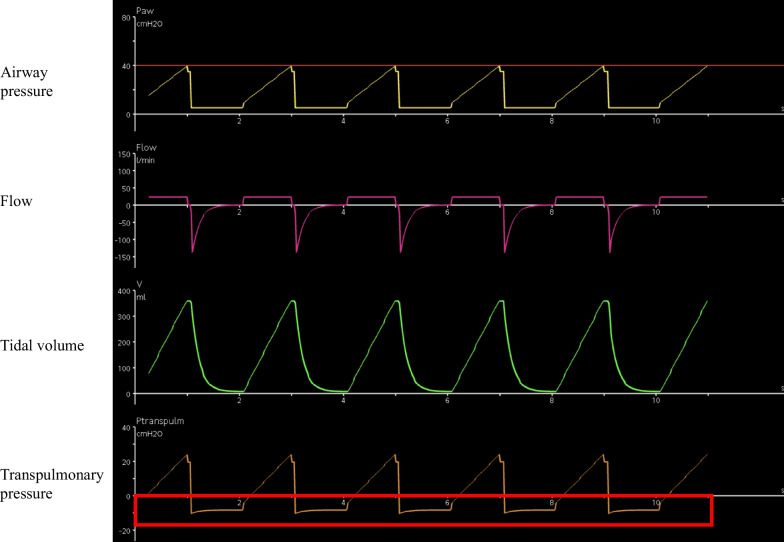
Negative expiratory transpulmonary pressure. The area surrounded by the red line indicates the negative expiratory transpulmonary pressure that may collapse the alveoli

Dorsal lung collapse occurs frequently in patients with ARDS, and the degree of collapse varies according to pleural pressure and lung compliance [13]. Conditions that decrease chest wall compliance (edema, posterior spinal scoliosis, increased intra-abdominal pressure) or shift the thoracic pressure capacity curve to the right (e.g., obesity) may result in elevated pleural pressure. Under these circumstances, the pleural pressure at end-expiration is often higher than the alveolar pressure at end-expiration, resulting in negative P_L_ at end-expiration, which can cause alveolar collapse [14] (Fig. 5). Increasing alveolar pressure at end-expiration through the application of PEEP can potentially prevent this collapse.

### Inspiratory effort

The respiratory muscle activity associated with spontaneous breathing produces a negative change in Ppl. Since Pes is a surrogate for Ppl, Pes can be used to estimate the pressure generated by the respiratory muscles (Pmus). Pmus is calculated as the difference between Pes and chest wall recoil pressure (Pcw) at a given tidal volume and is typically slightly higher than the change in Pes during tidal breathing (delta Pes). The magnitude of inspiratory effort can be quantified from several indices using Pes or Pmus, including the simplest and readily available delta Pes itself, as well as work of breathing (WOB), pressure time product (PTP), and pressure rate product (PRP) which will be discussed below.

## "How can it be used for clinical setting and what have previous studies shown”

### PEEP titration using transpulmonary pressure

Talmor et al. conducted a randomized controlled trial (EPVENT) in adult patients with acute respiratory failure, which targeted setting PEEP based on maintaining a positive P_L_ at end-expiration (direct method) versus using an empirical approach using the ARDS Network low PEEP/high F_I_O_2_ table [15]. The P/F ratio at 72 h was 88 mmHg higher in the esophageal pressure group than in the control group. Importantly, there was a trend for lower 28-day mortality in the esophageal pressure group. These results prompted a multicenter RCT, EPVENT-2 [16].

EPVENT-2 found no difference between the intervention and control groups with respect to the primary composite outcome of mortality and time off the ventilator or secondary endpoints such as mortality at 28 days and ventilator-free days. An important difference between EPVENT-2 included use of the ARDS Network high PEEP/low FiO2 table in the control group, instead of the low PEEP/high FiO2 table used in EPVENT-1. Post hoc analysis demonstrated heterogeneity in treatment effect based on severity of illness [17], as PEEP guided by the esophageal pressure was associated with lower mortality in patients with APACHE-II below the median value and may have had the opposite effect in patients with higher APACHE-II. Regardless of the treatment group or severity of multiorgan failure, mortality was lowest when PEEP titration brought *P*_*L*_ end-expiration to approximately 0 cm H_2_O. This highlights that while on a population level end-expiratory *P*_*L*_ aligns with the high PEEP/low FiO2 table, there is variation on an individual patient basis and esophageal pressure can help individualize PEEP management for a given patient, which may not be apparent with the PEEP/F_I_iO_2_ table.

A recent multicenter prospective observational study [18] again highlights potential advantages to *P*_*L*_ measurements in subsets of patients. Specifically, they found that PL end-expiration > 0 was associated with lower 60-day mortality in obese patients with a BMI greater than 30. This also reinforces the value of esophageal pressure measurements for PEEP titration, specifically for patients with impaired chest wall compliance.

### Extubation readiness

Assessment of patient effort is crucial in determining extubation readiness, and can be estimated using esophageal manometry [19]. Extubation failure (or weaning failure) is caused by an imbalance between respiratory load (respiratory work) and respiratory capacity (respiratory muscle strength), making it important to assess the total balance between respiratory load and capacity in the evaluation of ventilator liberation. Pi/Pimax is a measure of the balance between these two and is calculated as the ratio of change in the airway or esophageal pressure during inspiration over the maximum change in the inspiratory airway or esophageal pressure during occlusion. It has been reported that a high Pi/Pimax measured immediately after extubation is associated with reintubation in children [20]. A limitation of Pi/Pimax is that it does not capture the duration of time spent in inspiration, and does not isolate the diaphragm from other respiratory muscles. Tension-time index (TTI) is calculated as (Pdi/Pdimax)/(TI/Ttot) (Pdi: mean transdiaphragmatic pressure during inspiration (Pes-Pgastric), Pdimax: transdiaphragmatic pressure during maximum inspiration, TI: inspiration time, Ttot: time for one respiratory cycle). Previous studies have highlighted TTI of < 0.15 predicts respiratory muscle fatigue in adults and children [21–23].

### Upper airway obstruction (UAO)

**Fig. 6:**
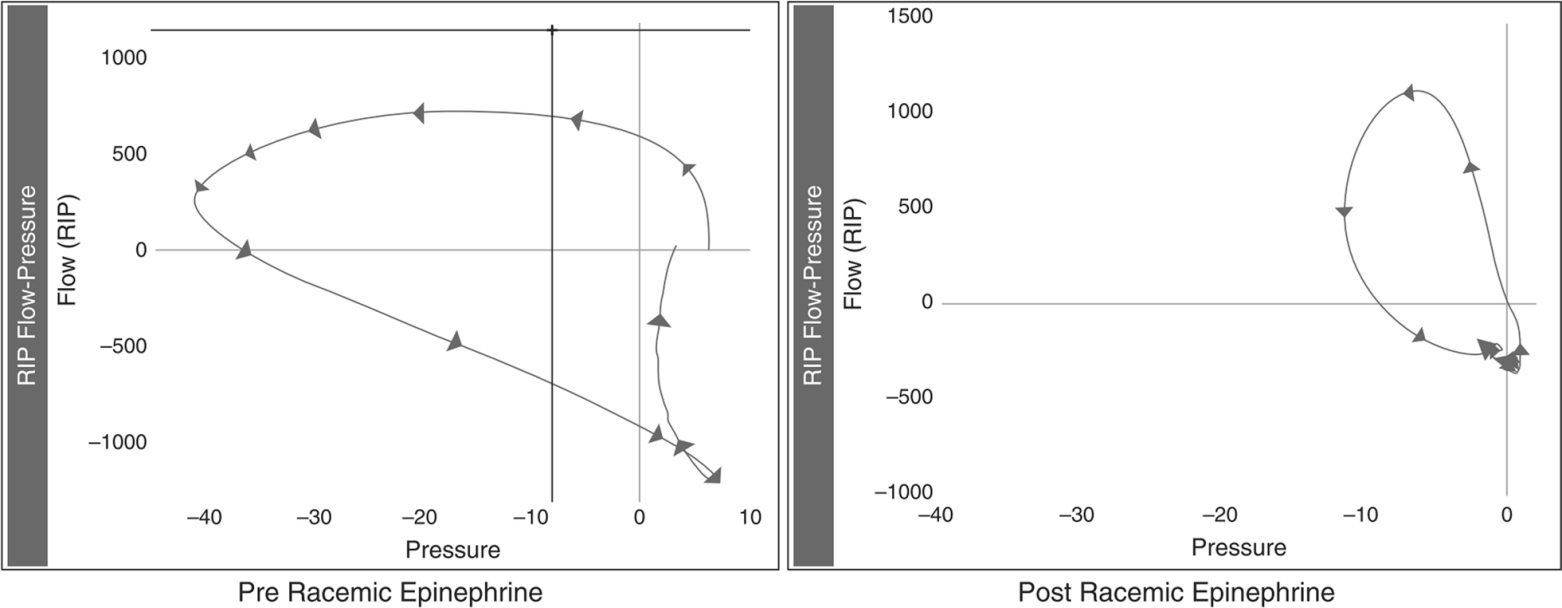
Example of a flow-limitation pattern in a patient with subglottic upper airway obstruction after extubation. Applying permission to reprint from [24] with permission of the American Thoracic Society. Example of subglottic upper airway obstruction data of an infant after extubation. The data indicate inspiratory flow limitation (left), that is, no increase in flow despite a continual decrease in esophageal pressure. A significant improvement was observed 20 min after racemic epinephrine administration (right). Esophageal pressure was measured in centimeters of water. RIP = respiratory inductance plethysmography. Copyright © 2022 American Thoracic Society. All rights reserved. Cite: Khemani RG, Hotz J, Morzov R, et al. 2016 Evaluating Risk Factors for Pediatric Post-extubation Upper Airway Obstruction Using a Physiology-based Tool. Am J Respir Crit Care Med. 193:198–209. The American Journal of Respiratory and Critical Care Medicine is an official journal of the American Thoracic Society. Example of subglottic upper airway obstruction data of an infant after extubation. The data indicate inspiratory flow limitation (left), that is, no increase in flow despite a continual decrease in esophageal pressure. A significant improvement was observed 20 minutes after racemic epinephrine administration (right). Esophageal pressure was measured in centimeters of water. *RIP* respiratory inductance plethysmography

Esophageal manometry has also been used as a tool to detect upper airway obstruction after extubation in children when combined with respiratory inductance plethysmography. The diagnosis of UAO is made with inspiratory flow limitation, characterized by disproportionately high inspiratory effort (negative esophageal pressure) relative to the change in flow (Fig. 6) [24]. The authors identified that EM with respiratory inductance plethysmography could provide objective warning signs indicating UAO earlier than the bedside clinician's subjective assessment, and is particularly useful in children, where UAO is a frequent cause of failed extubation.

### Ventilator-induced diaphragm dysfunction (VIDD) and P-SILI

The preservation of spontaneous breathing has many advantages during critical illness. Contraction of the diaphragm by spontaneous breathing distributes ventilation to areas of better perfusion in the dorsal lungs compared with ventilation under neuromuscular blockade [25]. Spontaneous breathing during ventilation may improve gas exchange, maintain peripheral muscles, and prevent diaphragm atrophy [26, 27]. Subphysiological levels of patient effort, often from over assistance from the ventilator, are a major risk factor for ventilator-induced diaphragm dysfunction (VIDD). VIDD has been associated with prolonged mechanical ventilation, reintubation, functional impairment, and mortality [28–31]. Over-assistance is reported to occur frequently in both adult and pediatric ventilated patients [32, 33]. In contrast, excessive spontaneous respiratory effort leads to increased pulmonary stress and strain, increased pulmonary perfusion, and patient–ventilator asynchrony, resulting in lung injury known as effort-dependent lung injury or patient self-inflicted lung injury (P-SILI) [3, 27]. P-SILI has been shown to be associated with multiple organ dysfunction, progression of lung injury, and death [3, 27, 34].

It has recently become a therapeutic target to maintain patient effort at physiological levels to balance the risks of P-SILI against the risks of VIDD. A recent consensus conference of experts on adult and pediatric ventilation emphasized the importance of balancing protective ventilation of the lung and diaphragm in ARDS [29]. The principle is that lung-protective ventilation is the first priority because of the solid evidence that ventilator-induced lung injury is harmful; however, whenever possible, treatment goals should consider the risk of P-SILI and VIDD and try to maintain patient effort at physiologic levels. Currently, these principles are being tested in clinical trials [35]. Therefore, it is important for bedside physicians to be able to assess the degree of respiratory effort required to make informed ventilator management decisions. Esophageal manometry represents the accepted standard for estimating patient effort or work of breathing.

### Work of breathing (WOB)

**Fig. 7:**
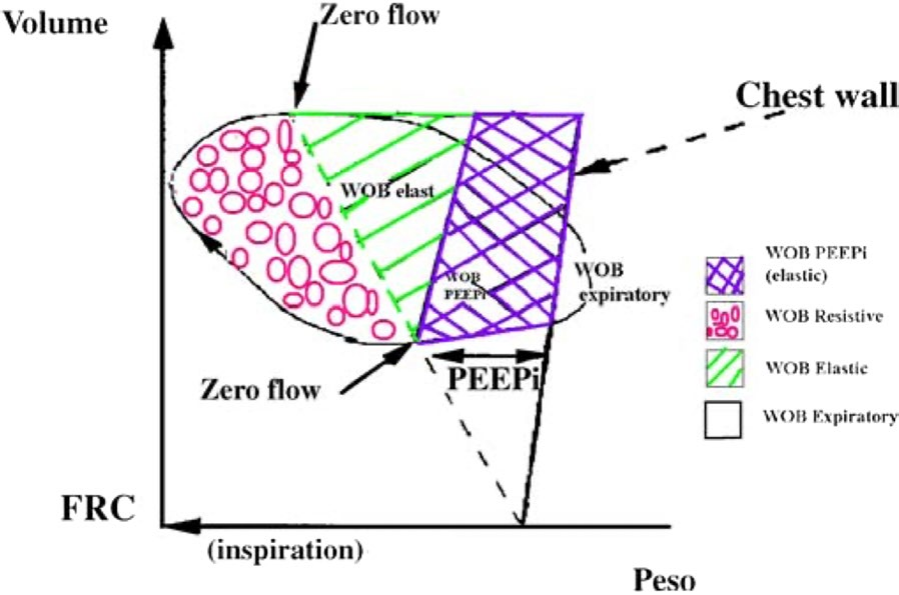
Method of calculating WOB. Permitted to reprint from [36] with permission form Springer Nature. x-axis: esophageal pressure, y-axis: lung volume. Since the patient's Work of breathing (WOB) includes the work of expanding the thorax in addition to the work of expanding the lungs, WOB is the area bounded by the straight line whose slope is the chest wall compliance and the esophageal pressure (x-axis) and lung capacity (y-axis) plot curves in the inspiratory phase [21]

WOB can be calculated by plotting a curve of one respiratory cycle with esophageal pressure on the x-axis and lung capacity on the y-axis, and the area enclosed by a straight line whose slope is the chest wall compliance. (Fig. 7) [36]. This represents the most precise measure of the work being performed by the respiratory muscles, but has a disadvantage of requiring an accurate measure of volume, limiting application to invasively ventilated patients, for the most part.

#### Pressure time product (PTP)

**Fig. 8:**
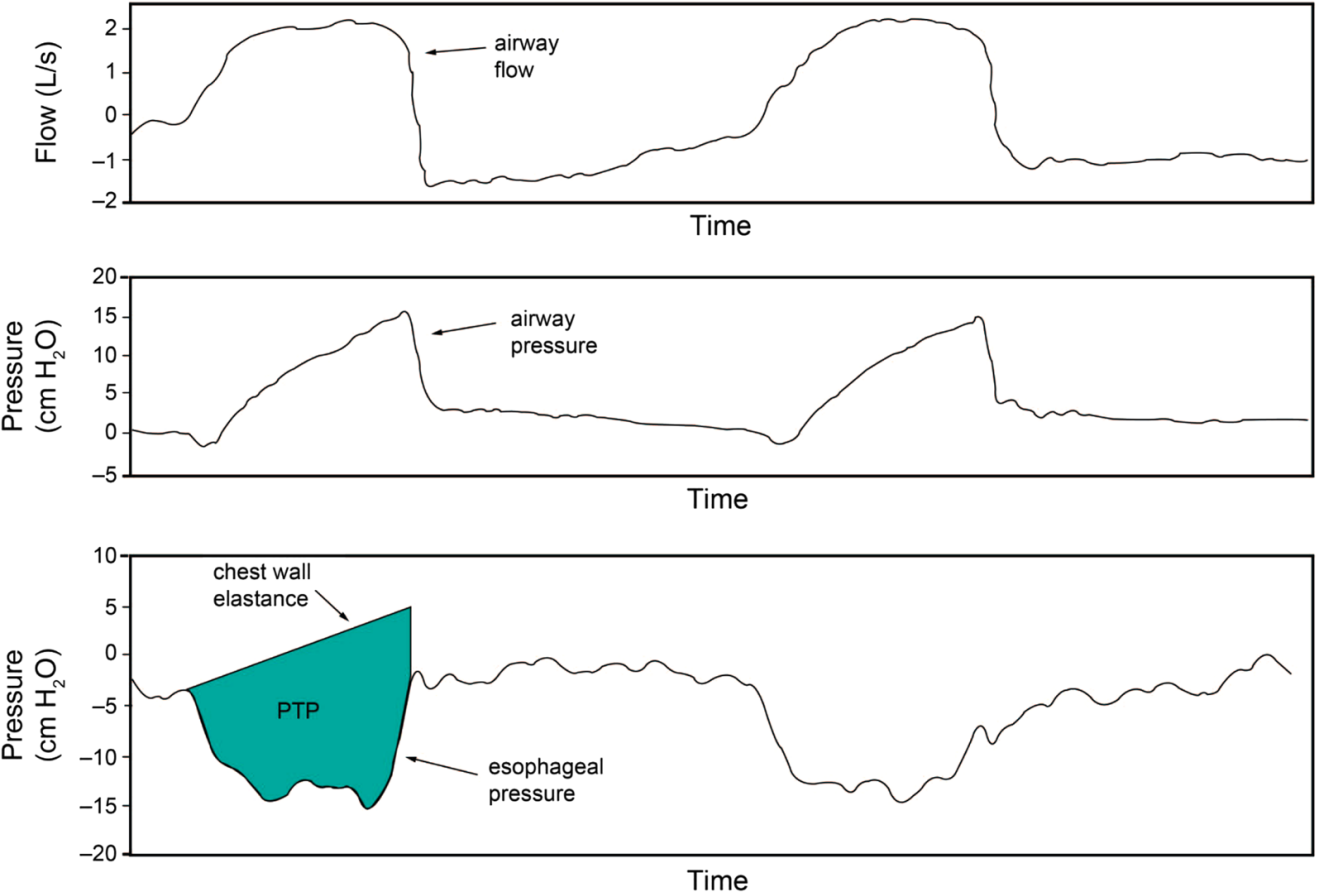
Method of calculating PTP. Applying permission to reprint from [21] with permission from Daedalus Enterprises; permission conveyed through Copyright Clearance Center, Inc. The upper curve is the time-flow curve, the middle curve is the time-airway pressure curve, and the lower curve is the time-esophageal pressure curve in the ventilated patient with spontaneous breathing. Pressure time product (PTP) can be calculated as the area bounded by the curve of the esophageal pressure in the negative direction during one inspiration (i.e., the pressure that inflates the lungs, color) and the straight line with thoracic compliance taken as the slope (i.e., the pressure that inflates the thorax), multiplied by respiratory rate

PTP is a measure of EOB, calculated as the time-based integral of Pmus, that is, the difference between the estimated recoil pressure of the chest wall calculated from the tidal volume and Ccw and Pes during inspiration (Fig. 8). PTP is commonly reported over a 1-min interval. It may be more strongly correlated with respiratory muscle oxygen consumption than with WOB [21, 37, 38]. In adults, the optimal PTP in ventilated patients remains an issue of debate, but suggested targets have been proposed at 50–150 cm H2O·s/min [39].

#### Pressure rate product (PRP)

PRP is a measure of breathing effort and is the product of the respiratory rate and the change in esophageal pressure during the respiratory cycle. PRP typically does not subtract the component related to chest wall elastance and has been used most extensively in children. It has the advantage of not requiring a measurement of volume or flow (unlike WOB and PTP), which makes it optimal for studying patients who may not be intubated. Using data measured both before and after extubation in children, a PRP in the range of 200–400 cmH2O/min is considered physiologic and has a low risk of reintubation [19, 40]. In children, this PRP range roughly corresponds to the proposed PTP range of 50–150 cm H2O·s/min for adult targets [19].

### Delta esophageal pressure (delta Pes)

Delta Pes is the difference between the lowest Pes value during inspiration and the Pes value just before the beginning of the inspiration. Of all the estimates of patient effort using esophageal manometry, it is the easiest to measure. Delta Pes targets have been suggested to be between − 2 cmH2O and  − 12 cmH2O [29]. Of note, delta Pes is a surrogate for Pmus, but does not correct for the elastic recoil of the chest wall.

Although there are still few reports on the clinical outcomes of maintaining respiratory effort in a target range, Phase I studies highlight the feasibility and possible improvement in clinical outcomes, such as time to the first SBT and more VFDs [41]. A randomized controlled trial in pediatric patients is currently ongoing [35], and one in adults is under development.

### Patient–ventilator asynchrony (PVA)

PVA is a major problem in ventilated patients and is related to a mismatch between the patient and the ventilator related to spontaneous breathing efforts. The frequency of PVA is estimated to be as high as 80% [42]. However, the impact of PVA on clinical outcomes in ventilated patients appears to be inconsistent across studies; Thille et al. reported that a higher incidence of PVA is associated with a longer duration of ventilation but not with increased mortality [43]. On the contrary, Blanch et al. found that patients with a higher incidence of PVA had a significantly higher mortality rate in the ICU than those with a lower incidence, but the duration of ventilation did not differ significantly between the two groups [44]. A systematic review and meta-analysis by Kyo reported that PVA might be associated with clinical outcomes; and that more attention should be paid to PVA [45].

**Fig. 9:**
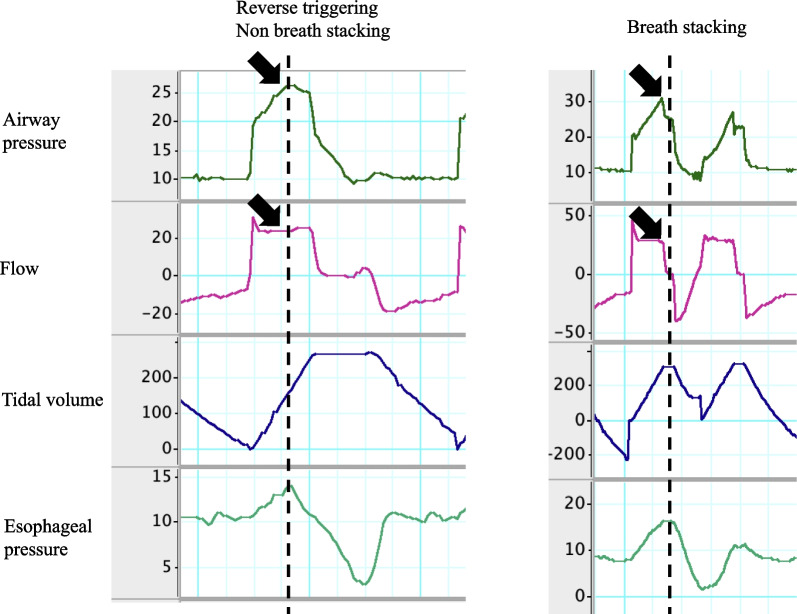
Reverse triggering waveforms. Waveforms of non-breath stacking (left) and breath stacking (right). Black arrows indicate airway pressure and flow at the initiation of spontaneous efforts. The breath on the left has reverse triggering, without a breath stack

Understanding PVA and its severity requires assessment of the patient’s effort. Esophageal manometry can serve as the gold standard method to characterize the timing, phase, magnitude, and duration of patient effort, all of which can contribute to different forms of PVA. For example, reverse triggering is a PVA subtype that is more clearly identified by esophageal manometry. Reverse triggering has been reported in animals and humans since the 1970s [46]. However, it was not until 2013 that this was reported as a ventilator asynchrony [47]. This occurs when the patient’s effort begins after lung inflation from a controlled breath. Recognizing the timing of spontaneous respiratory effort is very important for its diagnosis, and esophageal pressure can provide a direct measure of this timing (Fig. 9). The incidence of reverse triggering during mechanical ventilator management with acute respiratory failure may be as high as 40–50% in both adults and children [48, 49]. Reverse triggering may lead to double cycling (breath stacks) and contribute to lung injury; however, its clinical impact has not yet been fully elucidated.

## Summary

Esophageal manometry has several uses to facilitate ventilator management in both adults and children. These include measurement of transpulmonary pressure to minimize the risk of VILI or P-SILI, estimation of spontaneous respiratory effort to minimize the risks of VIDD, evaluation of extubation readiness, and identification of complications such as post-extubation upper airway obstruction and patient ventilator asynchrony. However, there are technical details related to the placement and calibration of esophageal balloon catheters which are important to consider when making clinical decisions. It is likely that some of these technical issues have limited clinical use; however, this tool represents an invaluable method for individualizing patient ventilator management.x-axis: esophageal pressure, y-axis: lung volume. Since the patient's Work of breathing (WOB) includes the work of expanding the thorax in addition to the work of expanding the lungs, WOB is the area bounded by the straight line whose slope is the chest wall compliance and the esophageal pressure (*x*-axis) and lung capacity (*y*-axis) plot curves in the inspiratory phase [21].

The upper curve is the time–flow curve, the middle curve is the time–airway pressure curve, and the lower curve is the time–esophageal pressure curve in the ventilated patient with spontaneous breathing. Pressure time product (PTP) can be calculated as the area bounded by the curve of the esophageal pressure in the negative direction during one inspiration (i.e., the pressure that inflates the lungs, color) and the straight line with thoracic compliance taken as the slope (i.e., the pressure that inflates the thorax), multiplied by respiratory rate.

## Data Availability

Not applicable.

## Abbreviations

VILI: Ventilator-induced lung injury
Ptp: Transpulmonary pressure
P-SILI: Patient self-inflicted lung injury
VIDD: Ventilator-induced diaphragm dysfunction
EOB: Effort of breathing
WOB: Work of breathing
PTP: Pressure time product
PRP: Pressure rate product
UAO: Upper airway obstruction
PVA: Patient–ventilator asynchrony
PEEP: Positive end-expiratory pressure
ARDS: Acute respiratory distress syndrome
BMI: Body mass index
APACHE-2: Acute Physiologic Assessment and Chronic Health Evaluation
